# Plasma Phospho-Tau-181 as a Diagnostic Aid in Alzheimer’s Disease

**DOI:** 10.3390/biomedicines10081879

**Published:** 2022-08-03

**Authors:** Ioanna Tsantzali, Aikaterini Foska, Eleni Sideri, Evdokia Routsi, Effrosyni Tsomaka, Dimitrios K. Kitsos, Christina Zompola, Anastasios Bonakis, Sotirios Giannopoulos, Konstantinos I. Voumvourakis, Georgios Tsivgoulis, George P. Paraskevas

**Affiliations:** 2nd Department of Neurology, School of Medicine, National and Kapodistrian University of Athens, “Attikon” General University Hospital, 12462 Athens, Greece; docjo1989@gmail.com (I.T.); dkfoska@gmail.com (A.F.); elenisideri1985@gmail.com (E.S.); evd.routsi@hotmail.com (E.R.); tsomaka@gmail.com (E.T.); dkitsos@icloud.com (D.K.K.); chriszompola@yahoo.gr (C.Z.); bonakistasos@med.uoa.gr (A.B.); sgiannop@uoi.gr (S.G.); cvoumvou@otenet.gr (K.I.V.); tsivgoulisgiorg@yahoo.gr (G.T.)

**Keywords:** Alzheimer’s disease, cerebrospinal fluid, plasma, biomarkers, phospho-tau

## Abstract

Cerebrospinal fluid (CSF) biomarkers remain the gold standard for fluid-biomarker-based diagnosis of Alzheimer’s disease (AD) during life. Plasma biomarkers avoid lumbar puncture and allow repeated sampling. Changes of plasma phospho-tau-181 in AD are of comparable magnitude and seem to parallel the changes in CSF, may occur in preclinical or predementia stages of the disease, and may differentiate AD from other causes of dementia with adequate accuracy. Plasma phospho-tau-181 may offer a useful alternative to CSF phospho-tau determination, but work still has to be done concerning the optimal method of determination with the highest combination of sensitivity and specificity and cost-effect parameters.

## 1. Introduction

Cerebrospinal fluid (CSF) levels of amyloid peptide β with 42 amino acids (Aβ_42_), tau protein phosphorylated at a threonine residue at position 181 (τ_P-181_) and total tau protein (τ_T_) constitute the three established (classical) biomarkers for Alzheimer’s disease (AD) [1]. They have been studied extensively during the last two decades and, with estimated sensitivities and specificities approaching or exceeding 90%, they have been incorporated in diagnostic criteria [2] and recommendations [3]. More recently, they have been considered as core features for the definition of AD as an in vivo biological process [4], regardless of the presence or absence of symptoms and their type or severity (mild cognitive impairment or dementia). They have proven to be useful as diagnostic tools for the diagnostic work-up of dementia [5,6,7,8] and some movement disorders [9,10] during life. Additional candidate CSF biomarkers, including α-synuclein [11,12] and the transactive response DNA binding protein-43 (TDP-43) [13], are being thoroughly investigated, but work still has to be done before they become established biomarkers.

Over the last few years, blood-based biomarkers for AD, especially the classical Aβ_42_, τ_P-181_ and τ_T_, have received much attention [14,15]. It has been observed that plasma biomarkers show changes almost simultaneously with CSF biomarkers, following similar trajectories [16]. Although the range of changes for plasma Aβ_42_ and τ_T_ is lower compared to CSF changes, it is similar for τ_P-181_ [16]. Thus, the later could serve as a surrogate biomarker for AD.

## 2. Why Plasma Biomarkers? Blood vs. CSF Sampling

Since the CSF is in close contact with extracellular/interstitial fluid, it is expected to reflect the biochemical changes occurring within the central nervous system with adequate accuracy and thus, it may be preferable to blood [17]. However, CSF sampling requires lumbar puncture (LP). It is a routine procedure in neurological wards, well-tolerated, with a very low incidence of complications, the most frequent being post-LP headache [18]. The use of atraumatic needles reduces the likelihood of headache [18] and, in dementia patients, a headache incidence of <4.5% has been repeatedly reported [19] even with the use of Quincke-type needles [20].

Despite the above, LP is a relatively (minimally) invasive procedure, rarely performed by non-neurologists, requiring hospitalization in some countries or institutions, and it is a source of concern or anxiety for some patients or relatives. Furthermore, the amount of CSF collected is not unlimited. On the other hand, blood sampling is a non-invasive, much more easy-to-perform and acceptable procedure, has no complications, requires no hospitalization, and it can be performed in outpatient wards or in the community, permitting the collection of a larger sample volume which, in turn, facilitates biochemical determination of a wider spectrum of analytes, whilst repeated venipuncture (if necessary for equivocal or conflicting results, for additional biochemical assessments or for follow-up) is far more easy and acceptable than repeated LP.

## 3. Plasma τ_P-181_ and Alzheimer’s Disease

Plasma τ_P-181_ levels significantly correlate with the cerebrospinal fluid levels [16] and with the Aβ and τ protein load in the cerebral parenchyma, according to studies using Positron Emission Tomography-scan [21] (Table 1). Plasma τ_P-181_ levels are 3.5-fold increased in patients with AD as compared to controls, and this change is greater than the one of any other plasma biomarker [16,21,22,23]. In asymptomatic individuals and in patients with mild cognitive impairment, increased plasma τ_P-181_ levels predict future transition to Alzheimer’s dementia [23], indicating that τ_P-181_ levels may become abnormal during the pre-dementia or even the presymptomatic stage of AD.

From the clinical point of view, plasma τ_P-181_ levels may show a significant diagnostic value, in order to discriminate Alzheimer’s disease from other neurodegenerative disorders, with an area under the curve (AUC) reaching 0.94–0.98 [23]. This discriminative value may prove useful for the differential diagnosis of AD from frontotemporal dementia [24], with an AUC at the level of 0.88 [22]. For the discrimination from vascular dementia AUC reaches 0.92, for the discrimination from progressive supranuclear palsy and corticobasal degeneration, AUC reaches 0.88, and for the discrimination from Parkinson disease or multiple system atrophy, AUC may reach 0.82 [25]. Furthermore, plasma τ_P-181_ may identify an additional AD pathology in patients with Lewy body diseases [26]. Based on the above, the diagnostic value of plasma τ_P-181_ may approach that of CSF τ_P-181_ [25], introducing the former as a promising surrogate biomarker for AD.

Plasma levels of τ_P-181_ may also have prognostic value, since they may predict cortical brain atrophy in AD [27], AD pathology at least 8 years prior to pathologic diagnosis [28] and progression to AD dementia even in presymptomatic subjects [29,30,31]. Indeed, longitudinal changes in plasma levels seem to correlate with the progression of the AD neurodegenerative process [32,33,34,35]. Recently, it has been suggested that τ_P-217_ may perform better than τ_P-181_ [31,32,36].

**Table 1:** The major conclusions of the latest studies concerning the role of plasma τ_P-181_ in the diagnosis of Alzheimer’s disease.

Conclusions	References
Plasma τ_P-181_ levels correlate with CSF levels	[16]
Plasma τ_P-181_ levels are significantly higher in AD patients compared to controls	[16,21,22,23]
Plasma τ_P-181_ levels may also increase in pre-symptomatic or mildly demented patients and serve as a possible predictive biomarker	[23,28,29]
Plasma τ_P-181_ levels may act as a discriminative biomarker between Alzheimer’s and other types of dementia	[24,25,27]

## 4. Comparison with Other Plasma Biomarkers

### 4.1. Beta Amyloid Levels

Shin et al. [37] had observed a statistically significant decrease of Aβ_42_ in the plasma of patients with Alzheimer’s disease, without alteration of Aβ_40_ as compared to the control group. However, the Aβ_42_/Aβ_40_ ratio made this difference even more conspicuous. Likewise, Janelidze et al. [38] observed a significant reduction of Aβ_42_ and Aβ_42_/Aβ_40_ in plasma, without change of Aβ_40_ levels. The findings of two other studies [39,40] were headed towards the same direction, showing statistically significant differences; however, the Aβ_42_/Aβ_40_ ratio (although greater than Aβ_42_ level alone) showed a moderate capacity to separate sporadic presenile Alzheimer’s disease cases from normal individuals, with an area under the Receiver Operating Characteristics curve reaching 0.76 and a sensitivity and specificity that did not exceed 70% [39], due to an adequate amount of overlapping values between Alzheimer’s disease and other groups [38,40]. Nonetheless, through the use of more developed and precise detection techniques (including multiplexed, densely aligned sensor array), the Aβ_42_/Aβ_40_ ratio may have the potential to reach a more compensatory capacity to separate Alzheimer’s disease from the control group with an area under the curve 0.925 and a sensitivity and specificity that accedes to 90% [41].

The plasma Aβ_42_/Aβ_40_ ratio seems to predict the amount of cerebral amyloid burden, irrespective of the presence of cognitive deterioration [40,42,43], a fact that could be useful for the early (pre-symptomatic) diagnosis of Alzheimer’s disease and the incorporation of pre-symptomatic patients in research for new medications. An abnormal Aβ_42_/Aβ_40_ ratio recognizes the presence of amyloid in cerebral parenchyma with an area under the curve reaching 0.88, and increasing to 0.94 with *APOE4* addition, whilst it recognizes the presence of increased cerebrospinal fluid levels of τ_P-181_ with an area under the curve reaching 0.85 [44]. In addition to these, diminished levels of Aβ_42_ are associated with decreased hippocampal volume and a higher risk of Alzheimer’s disease occurrence [45].

Not all studies are in agreement with the above data; Feinkohl et al. did not conclude to a statistically significant difference between Aβ_42_, Aβ_40_ and Aβ_42_/Aβ_40_ in the plasma of AD patients [46], while two other studies have found an increased plasma Aβ_42_ level as compared to the control group [47,48]. Most of the above studies use more advanced methodologies, like highly sensitive immunoassays, mass spectrometry, Simoa (single molecule array), Luminex xMAP^®^, ή IMR (immunomagnetic reduction). The use of those techniques is associated to a higher cost, regarding that the low-cost technical infrastructure of Enzyme-linked Immunosorbent Assay (ELISA), which is used for the measurement of classical cerebrospinal fluid biomarkers, cannot generally be reclaimed in the measurement of plasma biomarkers.

### 4.2. Total Tau Levels and Other Biomarkers

Despite some initial indications of reduction [49], the level of τ_T_ is elevated in the plasma of Alzheimer’s disease patients, although not significantly correlated to the cerebrospinal fluid level [50,51]. Nevertheless, an elevation of total tau protein has been observed in other disorders, including frontotemporal dementia [52], thus limiting the specificity of this biomarker, whose determination demands a Single Molecule Array (Simoa) assay.

Neurofilament light chain (NFL) level is another indicator of axonal damage that presents a significant increase in the plasma of Alzheimer’s disease patients [53] and in other neurodegenerative disorders; therefore, it consists of another sensitive but not specific biomarker [15].

The plasma level of α-synuclein, which is increased in Parkinson’s disease patients [54], would be considered as a suitable biomarker for the separation between Alzheimer’s disease and Lewy body synucleinopathies. However, there are several restrictions that require further research to estimate the diagnostic value of this biomarker [15]; those restrictions are mainly related to the nature of the molecule under determination (monomer or oligomeric protein, total, phosphorylated) and other pre-analytical factors.

## 5. Some Preanalytical Aspects

As with CSF collection and handling, pre-analytical aspects in plasma biomarkers determination (including τ_P-181_) may be extremely important for diagnostic accuracy. It seems that K2- or K3-EDTA is the preferable anticoagulant for blood collection [55]. Centrifugation should be performed within <1 h after blood collection (preferably < 30 min), followed by aliquoting in tubes filled to >75% of their volume and storage at −80 °C within 1 h from sampling [56,57,58,59,60]. Polypropylene should be the material of collecting and storage tubes. Those techniques and preanalytical protocols have been established by numerous study groups, including the Alzheimer’s Biomarkers Standardization Initiative. The conditions and temporal limits under which the blood sample is centrifuged and stored may affect the levels of tau protein and β-amyloid in the sample under test. Other anticoagulants, such as Li-heparin or Na-citrate, can dramatically reduce the levels of tau protein compared to K3-EDTA. In addition, a reduction in β-amyloid levels in a plasma sample separated after 6 hours compared to a freshly separated sample has been noted. Finally, the sequalae of freeze/thaw cycles are shown to minimally affect the levels of plasma biomarkers. It is therefore important that a sample is obtained, separated, and stored under conditions that do not affect the quality of results [56,57,58].

## 6. New Disease-Modifying Treatments and Plasma τ_P-181_

Among the various disease modifying treatments tested for AD, the monoclonal antibody aducanumab has been recently approved by the Food and Drug Administration in the USA (accelerated approval pathway) [60], but not by the EMA, while other monoclonal antibodies are currently under clinical trials. Although these antibodies act by removing brain parenchymal amyloid, they also lead to a decrease of CSF phospho-tau [61]. The latter may be used to monitor the biochemical treatment effect, although there is not necessarily a correlation between the efficacy of the drug and modification of the CSF biomarker levels. Plasma phospho-tau may prove a good alternative, allowing frequent biochemical follow up, more convenient to the patient compared to repeated lumbar punctures and less costly compared to repeated positron emission tomography for amyloid load. Indeed, new data from aducanumab trials indicate a significant decrease of plasma τ_P-181_ following treatment [62,63].

Furthermore, since disease-modifying treatments may be more effective at early stages of the disease, the diagnosis of AD during the preclinical stages by blood (and not CSF) sampling could open new perspectives in wide population screening.

## 7. Emerging Plasma τ_P-271_

The phosphorylation of tau proteins can emerge at multiple sites. Recent studies have shown an increased capacity of another phospho-tau protein, τ_P-271_, to discriminate patients between Alzheimer’s disease and other dementias. Studies on CSF levels of τ_P-271_ have shown to accurately discriminate amyloid-PET-positive from amyloid-PET-negative patients. Those promising findings have led to studies involving the accuracy of plasma levels of τ_P-271_ in early diagnosis of AD, alone or compared to τ_P-181_. Further studies are needed to determine the possible applications of this new biomarker and its contingent superiority upon τ_P-181_ [32,36,64].

## 8. Conclusions

It seems that plasma levels of τ_P-181_ may prove helpful (and probably better than other blood-based biomarkers) in AD diagnosis, and prediction of progression. The additional combined use of other plasma biomarkers may not offer advantage over τ_P-181_ alone. Furthermore, it may prove a useful tool for frequent biochemical follow-up of patients under disease-modifying treatments. Despite the above encouraging data, plasma biomarkers including τ_P-181_ cannot be considered as established biomarkers yet. There are still questions concerning the optimal method of determination, and some recent studies raise doubts about the diagnostic help of τ_P-181_, which may be lower compared to the value of other plasma biomarkers such as the combination of Aβ_42_ and neurofilament light chain (NFL). Still, much work has to be done, including extensive real-world studies, testing various combinations of plasma biomarkers and cost-effect analyses.

## Data Availability

Not applicable.
